# Application of postoperative autotransfusion in total joint arthroplasty reduces allogeneic blood requirements: a meta-analysis of randomized controlled trials

**DOI:** 10.1186/s12891-017-1710-2

**Published:** 2017-09-02

**Authors:** Weiping Ji, Xianfeng Lin, Ruoxia Zhang, Pan Tang, Jian Mo, Xinyi Teng, Qiuping Fan, Bo Wang, Shunwu Fan, Jianfeng Zhang, Shuai Chen, Kangmao Huang

**Affiliations:** 1 Department of Orthopaedic Surgery, Lishui City People’s Hospital, the Sixth Affiliated Hospital of Wenzhou Medical University, Lishui, China; 2 Department of Orthopaedic Surgery, Sir Run Run Shaw Hospital, Medical College of Zhejiang University, Hangzhou, China

**Keywords:** Postoperative autotransfusion, Total knee arthroplasty, Total hip arthroplasty

## Abstract

**Background:**

Total joint arthroplasty is associated with significant blood loss and often requires blood transfusion. However, allogeneic blood transfusion (ABT) may lead to severe problems, such as immunoreaction and infection. Postoperative autotransfusion, an alternative to ABT, is controversial. We conducted a meta-analysis to evaluate the ability of postoperative autotransfusion to reduce the need for ABT following total knee arthroplasty (TKA) and total hip arthroplasty (THA).

**Methods:**

Systematic literature searches for randomized controlled trials were performed using PubMed, Embase, and the Cochrane Library until February 2016. Relative risks (RRs) and weighted mean differences with 95% confidence intervals (CIs) were calculated using fixed-effect or random-effect models; we also evaluated publication bias and heterogeneity.

**Results:**

Seventeen trials with a total of 2314 patients were included in the meta-analysis. The pooled RRs of ABT rate between autotransfusion and the regular drainage/no drainage groups for TKA and THA were 0.446 (95% CI = 0.287, 0.693; *p* < 0.001) and 0.757 (95% CI = 0.599, 0.958; *p* = 0.020), respectively. In the subgroup analysis performed in TKA patients according to control interventions, the pooled RRs were 0.377 (95% CI = 0.224, 0.634; *p* < 0.001) (compared with regular drainage) and 0.804 (95% CI = 0.453, 1.426, *p* = 0.456) (compared with no drainage). In the subgroup analysis performed for THA, the pooled RRs were 0.536 (95% CI = 0.379, 0.757, *p* < 0.001) (compared with regular drainage) and 1.020 (95% CI = 0.740, 1.405, *p* = 0.904) (compared with no drainage).

**Conclusions:**

Compared to regular drainage, autotransfusion reduces the need for ABT following TKA and THA. This reduction is not present when comparing autotransfusion to no drainage. However, the reliability of the meta-analytic results concerning TKA was limited by significant heterogeneity in methods among the included studies.

**Electronic supplementary material:**

The online version of this article (doi:10.1186/s12891-017-1710-2) contains supplementary material, which is available to authorized users.

## Background

Total knee arthroplasty (TKA) and total hip arthroplasty (THA) are major orthopedic surgeries. Both procedures are associated with significant blood loss, generally from bone osteotomies, severed muscles, periosteal bleeding, and some patient-related factors such as bleeding disorders and comorbidities [1, 2]. It is estimated that perioperative blood loss is between 1000 mL and 1500 mL in TKA and that 18–95% of patients require donor transfusions [3]. Following primary THA, the rate of allogeneic blood transfusion (ABT) can be as high as 30–40% [4, 5]. However, ABT can lead to infection with human immunodeficiency virus and hepatitis C [6], allergic reactions, anaphylaxis, hemolytic reactions, lung injury, or graft-versus-host disease [7]. Not only may these conditions undermine the success of the surgery, but can also result in death. Moreover, allogeneic blood resources are limited and expensive.

To establish stable postoperative hemoglobin (Hb) levels and to reduce the need for ABT, various alternatives to donor transfusion have been proposed. For example, there has been support for preoperative blood donation [8, 9], acute normovolemic hemodilution [10], erythropoietin injections [11], autologous transfusion systems [12–18], correction of preoperative anemia [19], and pharmacologic agents such as tranexamic acid [20]. The optimal combination of techniques for particular patients and cost-effectiveness remains a matter of debate.

Recently, the concept of reinfusing blood collected from drains following TKA and THA has gained the interest of orthopedists. Drains are used to prevent hematoma accumulation and decrease the possibility of prolonged wound healing and infection [21]. Recent studies have reported that the transfusion of autologous blood has no effect in the majority of cases, but some studies support the method [22–24]. Furthermore, the usefulness of autotransfusion is uncertain due to methodological difficulties, such as no formal power analysis for study size and significance level, different transfusion triggers, and different autotransfusion devices [23]. In order to clarify the issue, we performed a meta-analysis that evaluated ABT rate, postoperative Hb, and adverse effects after total joint arthroplasty when using autotransfusion drainage, and the use of regular drainage or no drainage.

## Methods

### Database search

A systematic review was performed in accordance with guidelines [25]. All holdings of PubMed, the Cochrane Library, and Embase were searched for relevant trials published until February 2016 using the following terms: (1) “autotransfusion,” “autologous blood transfusion,” “blood transfusion,” or “autologous transfusion”; (2) “total knee arthroplasty,” “total knee replacement,” “TKA,” “total hip arthroplasty,” “total hip replacement,” or “THA”; and (3) “postoperative,” or “post-operation.” Only English studies with full texts were included in the final analysis. We chose the most recent study if several publications reported on the same set of patients. The search strategy is provided in Additional file 1.

### Inclusion and exclusion criteria

**Fig. 1:**
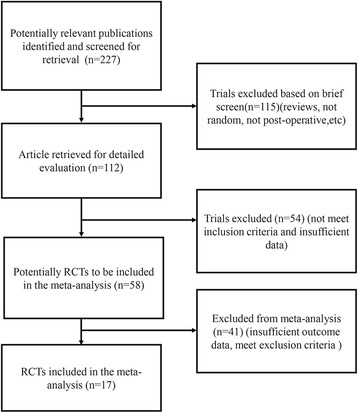
Flowchart for selecting studies

Eligible literature was carefully identified and selected according to the flow chart in Fig. 1. Studies were included on the basis of the following criteria: (1) a randomized controlled trial (RCT) was designed; (2) the comparison was between a postoperative autotransfusion system and no drainage/regular drainage (i.e., suction drainage, vacuum drainage, hemovac drainage); (3) the results included key data such as transfusion rate; and (4) patients underwent total joint replacement. The exclusion criteria were as follows: (1) an anticoagulant was added to the autotransfusion system; (2) patients underwent bilateral surgery or revision surgery; and (3) patients underwent TKA without a tourniquet because its use would have affected blood loss [26].

### Study selection and data extraction

Two authors (Weiping Ji and Xianfeng Lin) reviewed all titles and abstracts independently to determine which articles met the inclusion criteria. All variables and outcomes of interest were extracted independently. The authors solved disagreements by in-person discussion.

The study group referred to the autotransfusion group and the control group encompassed the regular drainage group and no drainage group. We extracted the following characteristics for all studies: year, country of study, type of surgery, population information (age, sample size and gender), autotransfusion systems, and funding sources. The outcomes included the numbers of patients needing allogeneic transfusion, postoperative Hb levels on day 1, day 2, day 3, and day 5, and adverse effects. In this meta-analysis, the transfusion rate was the primary outcome and defined as the rate of patients receiving allogeneic transfusion in both the study group and regular drainage/no drainage group postoperatively during hospitalization. The adverse events included complications such as wound infection and febrile reactions recorded in-hospital. Data relating to Hb levels and adverse effects were extracted to assess recovery after total joint arthroplasty. Blood loss volume, amount of blood transfusion per patient, length of hospital stay, and adverse reactions such as deep vein thrombosis (DVT) and swelling were extracted (data not shown), but the data were insufficient to be included in the meta-analysis.

### Validity assessment

The Cochrane Collaboration’s tool was used to assess the bias of all eligible RCTs [27]. The assessment criteria were as follows: (1) the method of randomization was adequate; (2) the treatment allocation was concealed; (3) the groups were similar in the most important prognostic indicators at baseline; (4) the patients were blinded to the intervention; (5) the caregivers were blinded to the intervention; (6) the outcome assessors were blinded to the intervention; (7) co-interventions were controlled; (8) compliance was acceptable in all groups; (9) the dropout rate was described and acceptable; (10) the timing of assessment in all groups was the same; and (11) intention-to-treat analysis was performed. We awarded a score of 1 for each item if it was completely met; scores of 0.5, 0, and N/A were awarded for partly met, not met, and unclear, respectively. The total score of each study was then calculated and the validity scores of >6 were considered to represent a low degree of bias.

### Statistical analysis

Statistical analyses were performed with STATA Version 12.0 (StataCorp LP, College Station, TX, USA). Relative risks (RRs) and corresponding 95% confidence intervals (CIs) were estimated by fixed-effect or random-effect meta-analyses. Weighted mean difference was used to perform meta-analysis for continuous outcomes. The significance of the pooled RRs was evaluated by a Z-test and *p* < 0.05. The I^2^ statistic was used to evaluate heterogeneity. A random-effect model was used when significant heterogeneity was detected among studies (*p* < 0.1, I^2^ > 50%). Otherwise, a fixed-effect model was adopted (I^2^ < 50%, *p* > 0.1). The fixed-effect model was adopted if a small number of studies (< 5) were included in the meta-analysis. Considering the source of heterogeneity, the studies were classified by control intervention. To explain some of the clinical heterogeneity observed in the included trials, a subgroup analysis by type of control intervention was performed for the primary outcome. Two sensitivity analyses were performed to assess the stability of pooled effects for TKA and THA. The RRs calculated after excluding a single study were compared with initial effects (before excluding). The results could be considered stable if the RRs did not change significantly (not beyond the initial CI). Egger’s test and Begg’s test were used to test for publication bias by assessing the association between intervention effects and a measure of study size. Egger’s test used actual standardized effect size while Begg’s test used ranks of effect sizes and variances. Funnel plots were used to depict asymmetry on visual inspection. A value of >0.05 for Pr > |z| (continuity correct) suggested no publication bias [28]. All *p-*values were two-sided.

## Results

### Study characteristics

Seventeen RCTs [2, 7, 12–18, 22–24, 29–33] were assessed and 2314 patients were included in the meta-analysis (Table 1). Sample sizes ranged from 36 to 231. Three studies included two control groups [13, 18, 33]. Another three studies included two types of surgery: TKA and THA [2, 17, 23]. The criteria for transfusion thresholds were varied and based on authors’ experiences, anesthetists’ protocols, or hospital policies (data not shown). The data of tranexamic acid use were rarely provided in the included studies. All of the included studies were published in 1992 or later. Validity scores are shown in Table 2 and two studies with validity scores of <6 were considered as having a high degree of bias [16, 33].

**Table 1:** Characteristics of the studies included in the meta-analysis

								Study group		Control group	
Study	Year	Country	Type of surgery	Sample size	Age	Men%	transfusion threshold (Hb)	Patients number (n)	Autotransfusion system	Patients number (n)	Treatment
Heddle [12]	1992	Canada	TKA	79	70.16	35.4	<9 g/dl	39	Solcotrans	40	Regular drain
Adalberth [13]	1998	Sweden	TKA	73	72	45.6	<9 g/dl	24	Solcotrans	25 24	Regular drain No drain
Thomas [14]	2001	UK	TKA	231	69.63	42.9	<9 g/dl	115	Cell Saver 5 Haemonetics	116	Regular drain
Cheng [15]	2005	Hong Kong	TKA	60	70.53	30	<9 g/dl	26	DONOR	34	Regular drain
Dramis [29]	2006	UK	TKA	49	70.04	30.61	<9 g/dl	32	CellTrans	17	Regular drain
Abuzakuk [30]	2007	UK	TKA	104	68.5	41.34	<9 g/dl	52	Bellovac	52	Regular drain
Moonen [17]	2007	UK	TKA THA	77 83	69.25	14.38	ASA classification	45 35	Bellovac, AstraTech AB Bellovac, AstraTech AB	32 48	Regular drain Regular drain
Smith [16]	2007	UK	THA	158	74.54	48.1	<8 g/dl	76	ABTrans	82	Regular drain
Amin [31]	2008	UK	TKA	178	70.35	46.07	<8 g/dl	92	Bellovac	86	Regular drain
Atay [2]	2010	Turkey	THA TKA	36 41	59.33 66.76	33.33 21.95	<8 g/dl	17 20	Transolog Transolog	19 21	Regular drain Regular drain
Cheung [18]	2010	UK	THA	153	68.12	45.1	Made by the ward doctors	53	Bellovac	52 48	Regular drain No drain
Horstmann [32]	2012	Netherland	THA	100	68.8	27	ASA classification	50	Bellovac	50	No drain
Kleinert [33]	2012	Switzerland	THA	120	65.33	49.17	<8 g/dl	40	Bellovac	40 40	Regular drain No drain
Sarkanovic [7]	2013	Serbia	TKA	112	66.5	23.21	<8.5 g/dl	55	Cell Saver	57	Regular drain
Horstmann [24]	2014	Netherland	TKA	115	68.48	29.57	ASA classification	59	Bellovac	56	No drain
Thomassen [23]	2014	Netherland	TKA THA	165 219	68.9	25.58	<8 g/dl	78 116	Bellovac Bellovac	87 103	No drain No drain
Teetzmann [22]	2014	Norway	THA	161	73	37.94	clinical judgement	74	Sangvia	87	No drain

*TKA* total knee arthroplasty, *THA* total hip arthroplasty

**Table 2:** Risk of bias in included studies

Study	Year	A	B	C	D	E	F	G	H	I	J	K	Total score
Heddle [12]	1992	1	1	1	0	0	N/A	1	1	N/A	1	1	7
Adalberth [13]	1998	1	1	0.5	1	1	N/A	1	1	N/A	1	1	8.5
Thomas [14]	2001	1	1	0	0.5	1	N/A	1	1	N/A	1	1	7.5
Cheng [15]	2005	1	1	0.5	1	0	N/A	0.5	1	N/A	1	1	7
Dramis [29]	2006	1	N/A	0.5	N/A	N/A	N/A	1	1	0.5	1	1	6
Abuzakuk [30]	2007	1	1	0.5	0.5	0.5	N/A	1	N/A	N/A	1	1	6.5
Moonen [17]	2007	1	1	1	N/A	N/A	N/A	1	1	0.5	1	1	7.5
Smith [16]	2007	1	1	0.5	N/A	N/A	N/A	1	N/A	N/A	1	1	5.5
Amin [31]	2008	1	1	0.5	1	1	N/A	1	N/A	N/A	1	1	7.5
Atay [2]	2010	1	1	0.5	N/A	N/A	N/A	1	1	N/A	1	1	6.5
Cheung [18]	2010	1	1	0.5	N/A	0	N/A	1	1	N/A	1	1	6.5
Horstmann [32]	2012	1	1	0.5	1	0.5	N/A	1	N/A	N/A	1	1	7
Kleinert [33]	2012	1	N/A	0.5	N/A	N/A	N/A	1	N/A	N/A	1	1	4.5
Sarkanovic [7]	2013	1	1	1	N/A	N/A	N/A	1	N/A	N/A	1	1	6
Horstmann [24]	2014	1	1	0	0.5	1	N/A	1	1	N/A	1	1	7.5
Thomassen [22]	2014	1	1	0.5	N/A	N/A	1	1	1	N/A	1	1	7.5
Teetzmann [23]	2014	1	N/A	0.5	N/A	N/A	N/A	1	1	1	1	1	6.5

*RCT* randomized controlled trial, *N/A* not clear

Eleven Cochrane Back Review Group criteria: (A) the method of randomization was adequate; (B) the treatment allocation was concealed; (C) the groups were similar in the most important prognostic indicators at the baseline; (D) the patients were blinded to the intervention; (E) the caregivers were blinded to the intervention; (F) the outcome assessors were blinded to the intervention; (G) co-interventions were controlled; (H) compliance was acceptable in all groups; (I) the dropout rate was described and acceptable; (J) the timing of assessment in all groups was the same; and (K) intention-to-treat analysis was performed

### Meta-analysis findings for TKA

**Fig. 2:**
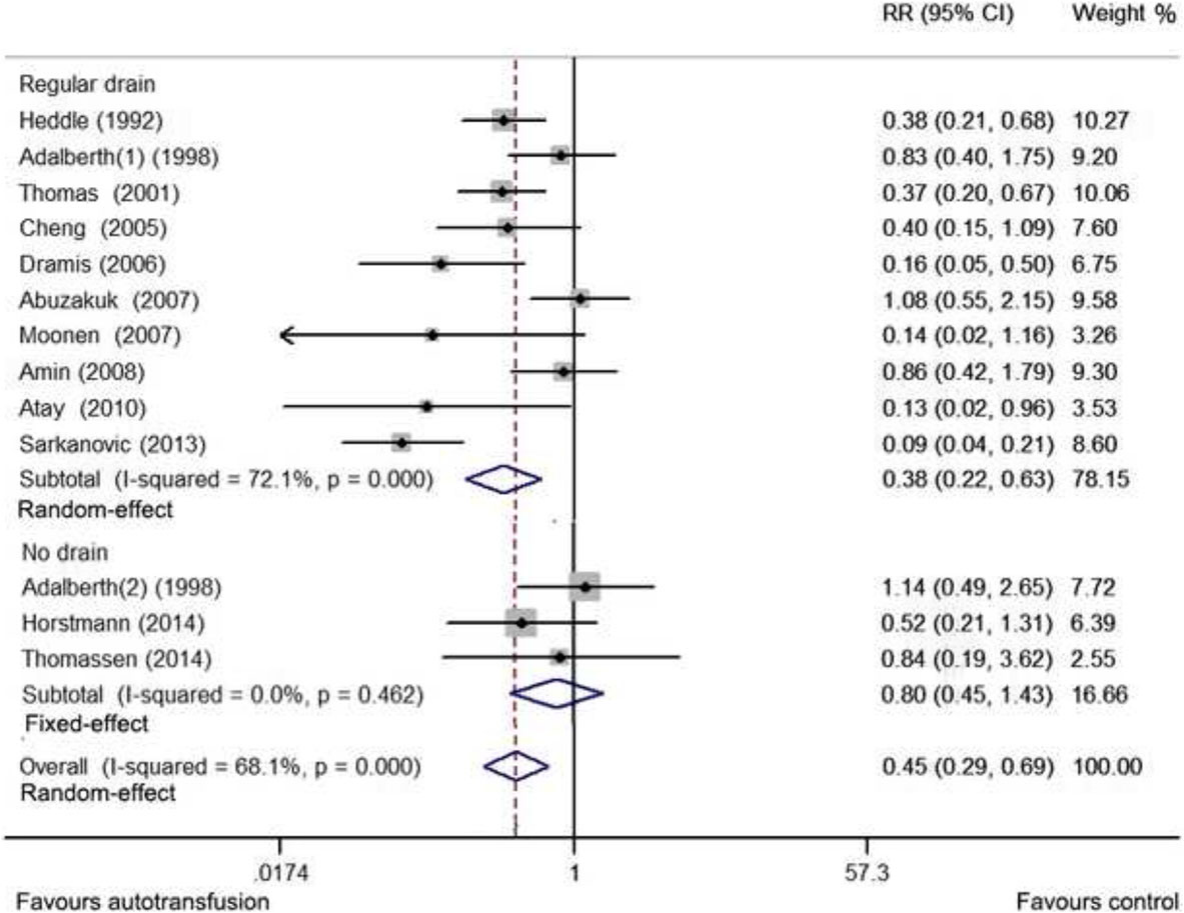
Relative risk (RR) of postoperative ABT requirements in TKA. ABT, allogeneic blood transfusion; TKA, total knee arthroplasty

The autotransfusion group had a significantly lower requirement for postoperative ABT (*p* < 0.001; RR = 0.446 [95% CI = 0.287, 0.693]; *I* ^2^ > 50%) than the regular drainage/no drainage group (Fig. 2). Although the autotransfusion group did not show superior reductions in ABT requirements when compared with the no-drainage group (*p* = 0.456; RR = 0.804 [95% CI = 0.453, 1.426]; *I* ^2^ < 30%), a significant reduction in ABT requirements was noted when compared with the regular drainage group (*p* < 0.001; RR = 0.377 [95% CI = 0.224, 0.634]; *I* ^2^ > 50%). The sensitivity analyses revealed stable results (Additional file 2: Figure S1), namely that the excluded studies did not influence the pooled RRs. The pooled results of post-operation Hb level (four studies included) suggested that the autotransfusion group had higher post-operative Hb levels than the regular drainage/no drainage group (Additional file 3: Figure S2). Six studies reported wound infections (three studies included) and febrile reactions (three studies included). We found no significant differences between the autotransfusion group and the regular drainage/no drainage group (Additional file 4: Figure S3). The reliability of these results was limited by the small number of studies included (< 5).

### Meta-analysis findings for THA

**Fig. 3:**
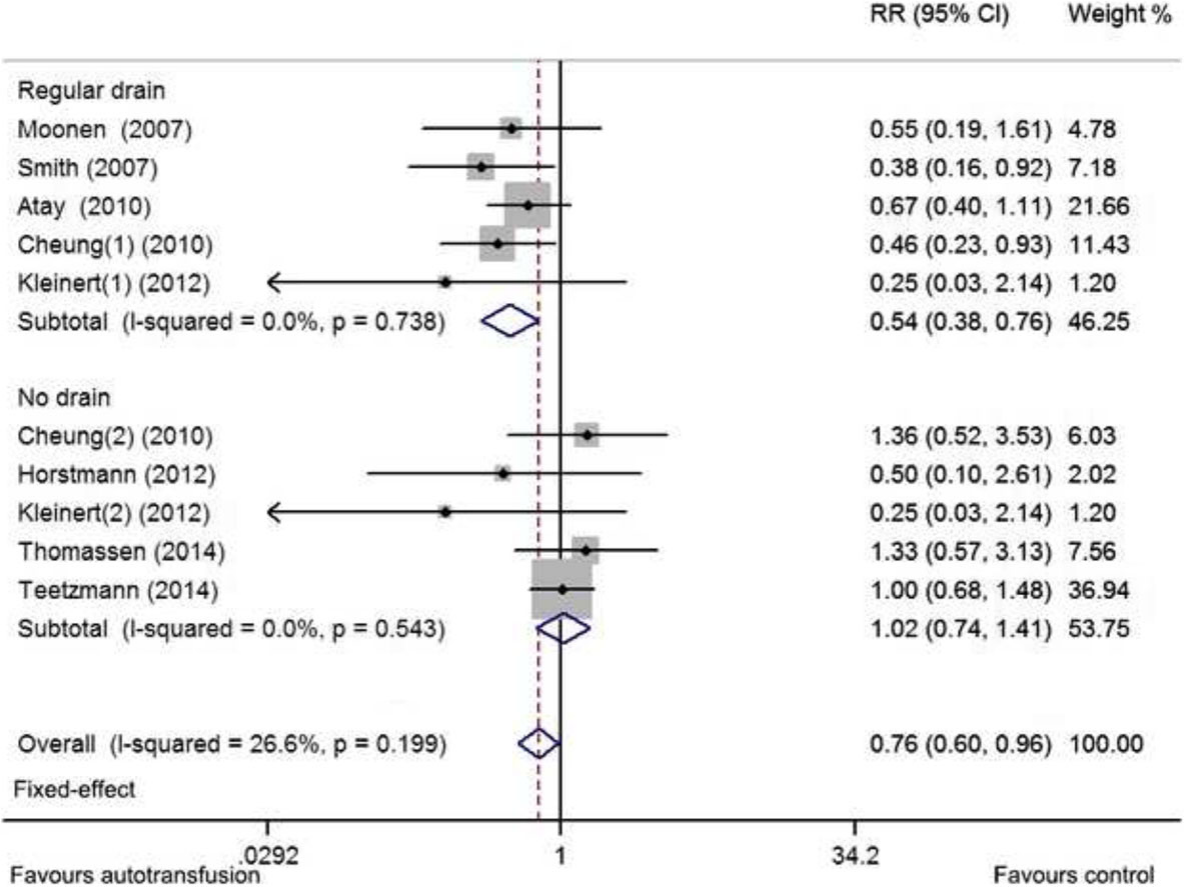
Relative risk (RR) of postoperative ABT requirements in THA. ABT, allogeneic blood transfusion; THA, total hip arthroplasty

The autotransfusion group had a significantly reduced need for postoperative ABT (*p* = 0.020; RR = 0.757 [95% CI = 0.599, 0.958]; *I* ^2^ < 30%) than the regular drainage/no drainage group (Fig. 3). Similar results were found when the autotransfusion group was compared with the regular drainage group and no-drainage groups. Specifically, ABT requirements significantly decreased when the autotransfusion group was compared with the regular drainage group (*p* < 0.001; RR = 0.536 [95% CI = 0.379, 0.757]; *I* ^2^ < 30%). However, the autotransfusion group did not demonstrate a significant reduction in ABT requirements compared with the no-drainage group (*p* = 0.904; RR = 1.020 [95% CI = 0.740, 1.405]; *I* ^2^ < 30%). Sensitivity analyses proved the results to be stable (Additional file 5: Figure S4). The autotransfusion group also had higher Hb levels (two studies included) compared with the regular drainage/no drainage group (Additional file 6: Figure S5). The pooled results for adverse effects (four studies reporting wound infections and two studies reporting febrile reactions) showed no significant differences between the groups (Additional file 7: Figure S6). The small number of included studies (< 5) limited the reliability of these results.

### Publication bias

Egger’s and Begg’s tests suggested a lack of publication bias with respect to ABT rate (Additional file 8: Table S1). According to this analysis, the included studies were relatively comprehensive and yielded statistically reliable results. The funnel plots did not reveal obvious asymmetry of the included studies (Additional file 9: Figure S7 and Additional file 10: Figure S8).

## Discussion

We conducted a meta-analysis to determine the effectiveness and safety of postoperative autotransfusion for TKA and THA. The findings revealed that postoperative autotransfusion could significantly reduce the number of patients who require postoperative ABT for TKA and THA compared with patients who receive regular drainage. However, the effect was not sustained when comparing autotransfusion with no drainage. The autotransfusion group had higher Hb levels than the regular drainage/no drainage group, and no significant difference in adverse reactions was observed between the two groups.

Although our results were positive for autotransfusion, the heterogeneity of the RRs for ABT rate in TKA was a limitation of the meta-analysis. Thus, the reliability of our meta-analytic estimates concerning TKA is questionable due to significant heterogeneity in methodologies. To reduce this heterogeneity in future trials, factors such as the type of system used, Hb triggers, comparison groups, and anesthesia methods should be taken into account. With this in mind, we performed subgroup and sensitivity analyses of the transfusion rates in TKA and THA. We believe that the variability in autotransfusion systems was an important consideration as at least seven autotransfusion systems were used in the 17 RCTs. Moreover, the skill of the operator performing blood reinfusion varied, which could have caused the differences in the volumes of re-infused blood among the studies. Insufficient data from existing RCTs limited our ability to analyze the effects of the different systems.

A previous meta-analysis drew different and incomplete conclusions regarding the benefits of autotransfusion. Zhao et al. and Markar et al. showed that autotransfusion drainage effectively reduced the demand for ABT after total joint arthroplasty when compared with regular drainage [34, 35]. However, Li et al. found no statistically significant differences in ABT rate between autotransfusion drainage and no/regular drainage in THA [36], which could be explained by the fact that only one study was included in the comparison [33].

Our meta-analysis revealed some distinct differences from the findings of previous studies. First, in this study, strict inclusion and exclusion criteria were observed in order to omit studies that implemented anticoagulants in the autotransfusion system [37, 38] or were performed without a tourniquet. Anticoagulants and tourniquets would affect blood loss during total joint arthroplasty. Second, two new RCTs were published [22, 23], neither of which showed a significant effect of the autotransfusion system compared with no drainage. In addition, few systematic reviews have considered the differences between autotransfusion drainage and no drainage for both TKA and THA. Finally, our research synthetically analyzed all related studies and gathered comprehensive results regarding the effects of autotransfusion in patients who underwent total joint arthroplasty.

The most important finding of this meta-analysis was that there was no significant difference in ABT rate between autotransfusion drainage and no-drainage. Recently, Thomassen et al. examined 575 patients who underwent primary hip and knee replacement with autotransfusion or no drainage [23]. The authors did not identify any significant difference in the need for ABT between the groups, which is in accordance with our results and those of many other studies (e.g., [13, 18, 22, 24, 32, 33]. Although the autotransfusion group had a significant reduction of ABT rate compared with the regular drainage/no drainage group in our meta-analysis, it is clear that autotransfusion drainage and no drainage have similar effects on ABT reduction.

To date, it remains controversial whether autotransfusion is superior to regular drainage. Atay et al. and Moonen et al. reported that the need for ABT was decreased by autotransfusion in both TKA and THA [2, 17]. Heddle et al. and others reported the same results for TKA alone [12, 14]. In contrast, some studies have shown that the use of an autotransfusion system fails to reduce the need for ABT after TKA [30, 31] or THA [18, 33]. This meta-analysis identified a statistically significant reduction in ABT requirements in patients who underwent autotransfusion drainage when compared with those who received regular drainage following TKA or THA. The previous meta-analyses performed by Markar et al. and Zhao et al. reported the same results for TKA alone [34, 35]. Other meta-analyses also compared regular drainage and no drainage for TKA [39] and THA [40], but there was no support for regular drainage in these reports. In conclusion, autotransfusion drainage and no drainage were superior to regular drainage. The mechanisms underlying these results should be explored in the future.

Adverse reactions including wound infection and febrile reactions were similar in the two groups, which mirrors the findings of previous meta-analyses [35, 36]. However, significant differences were noted by Zhao et al. between autotransfusion drainage and suction drainage in TKA with regard to febrile reactions [34]. This echoes Soosman’s report [41], which suggested a higher rate of febrile reactions than previous reports. Considering the low incidence of febrile reactions and wound infections in our results, we can only infer that autotransfusion drainage had a similar degree of safety when compared with regular drainage/no drainage. Notably, the number of included studies was small, limiting the reliability of these results. Further studies with sufficient data are required to evaluate the safety of autotransfusion systems.

We did not pool the data regarding blood loss volume, amount of blood transfusion per patient, length of hospital stay, and adverse reactions such as DVT and swelling because of insufficient data. Most RCTs stated the protocol for transfusion triggers, but whether strict transfusion rules were followed remains unknown. In addition, the transfusion triggers were not identical among the different RCTs. Thus, a subgroup analysis of transfusion triggers was not appropriate.

This meta-analysis has some limitations. First, the reliability of pooled results concerning TKA is limited by significant heterogeneity in methodological approaches. Second, the number of studies including data related to secondary outcomes was small. Thus, further meta-analyses including more studies and more information on safety outcomes are required in the future. Third, the results were based on many unadjusted factors. A more precise analysis should be conducted that allows for the time of randomization, drain insertion time, timing of drain opening and closing, and financial factors. Finally, the protocol for the systematic review was not prospectively registered. Thus, the transparency of our approach cannot be ascertained.

## Conclusion

Autotransfusion systems reduce the need for ABT compared with regular drainage, but this reduction is not maintained in comparison to procedures that employ no drainage. The use of autotransfusion is characterized by a reduced need for ABT, higher Hb levels, and similar adverse reactions when compared with control groups (regular drainage and no drainage). However, the reliability of our assessment of secondary outcomes was limited by the inclusion of a small number of studies including these data. Transfusion triggers and the operation procedure should be explored in future study.

## Additional files

Additional file 1: Search strategy. (DOCX 14 kb)

Additional file 2: Figure S1. Sensitivity analysis of postoperative ABT requirements in TKA. ABT, allogeneic blood transfusion; TKA, total knee arthroplasty. (TIFF 106 kb)

Additional file 3: Figure S2. Weighted mean differences (WMDs) of postoperative hemoglobin (Hb) level in TKA. TKA, total knee arthroplasty. (TIFF 190 kb)

Additional file 4: Figure S3. Relative risk (RR) of postoperative adverse reactions in TKA. TKA, total knee arthroplasty. (TIFF 82 kb)

Additional file 5: Figure S4. Sensitivity analysis of postoperative ABT requirements in THA. ABT, allogeneic blood transfusion; THA, total hip arthroplasty. (TIFF 84 kb)

Additional file 6: Figure S5. Weighted mean differences (WMDs) of postoperative hemoglobin (Hb) level in THA. THA, total hip arthroplasty. (TIFF 124 kb)

Additional file 7: Figure S6. Relative risk (RR) of postoperative adverse reactions in THA. THA, total hip arthroplasty. (TIFF 78 kb)

Additional file 8: Table S1. Publication bias determined using the Egger and Begg’s tests. (DOCX 18 kb)

Additional file 9: Figure S7. Funnel plot of included studies for TKA. TKA, total knee arthroplasty. (TIFF 40 kb)

Additional file 10: Figure S8. Funnel plot of included studies for THA. THA, total hip arthroplasty. (TIFF 39 kb)

Additional file 11: **Dataset.** The dataset supporting the conclusions of this article. (XLSX 14 kb)

Additional file 12: Table S2. The transfusion thresholds and funding source of included studies. (DOCX 87 kb)

## Abbreviations

ABT: Allogeneic blood transfusion
CIs: Confidence intervals
DVT: Deep vein thrombosis
Hb: Hemoglobin
PRISMA: Preferred Reporting Items for Systematic Reviews and Meta-Analyses
RR: Relative risk
THA: Total hip arthroplasty
TKA: Total knee arthroplasty
