# Supplementary material for: Application of postoperative autotransfusion in total joint arthroplasty reduces allogeneic blood requirements: a meta-analysis of randomized controlled trials
Source: BMC Musculoskelet Disord. 2017 Sep 2;18:378. doi: 10.1186/s12891-017-1710-2 (PMC5581423; doi:10.1186/s12891-017-1710-2)
Supplement: Supplementary file 1 — Search strategy. (DOCX 14 kb) [file 12891_2017_1710_MOESM1_ESM.docx]

**Search Strategy**

((((("Randomized Controlled Trials as Topic"[Mesh]) OR random*) OR Randomized Controlled Trial[Publication Type])) AND ((((((THA) OR TKA) OR total knee arthroplasty) OR total hip arthroplasty) OR "Arthroplasty, Replacement, Hip"[Mesh]) OR "Arthroplasty, Replacement, Knee"[Mesh])) AND (((("Blood Transfusion, Autologous"[Mesh]) OR autologous blood transfusion) OR autotransfusion) OR Autologous transfusion)

#1 Search random*

#2 Search "Randomized Controlled Trials as Topic"[Mesh]

#3 Search #1 or #2

#4 Search THA

#5 Search TKA

#6 Search total knee arthroplasty

#7 Search total hip arthroplasty

#8 Search Arthroplasty, Replacement, Hip"[Mesh]

#9 Search "Arthroplasty, Replacement, Knee"[Mesh]

#10 Search #4 or #5 or #6 or #7 or #8 or #9

#11 Search "Blood Transfusion, Autologous"[Mesh]

#12 Search autologous blood transfusion

#13 Search autotransfusion

#14 Search Autologous transfusion

#15 Search #11 or # 12 or #13 or #14

#16 Search #3 and #10 and #15
