# Supplementary figures and images for: Application of postoperative autotransfusion in total joint arthroplasty reduces allogeneic blood requirements: a meta-analysis of randomized controlled trials
Source: BMC Musculoskelet Disord. 2017 Sep 2;18:378. doi: 10.1186/s12891-017-1710-2 (PMC5581423; doi:10.1186/s12891-017-1710-2)

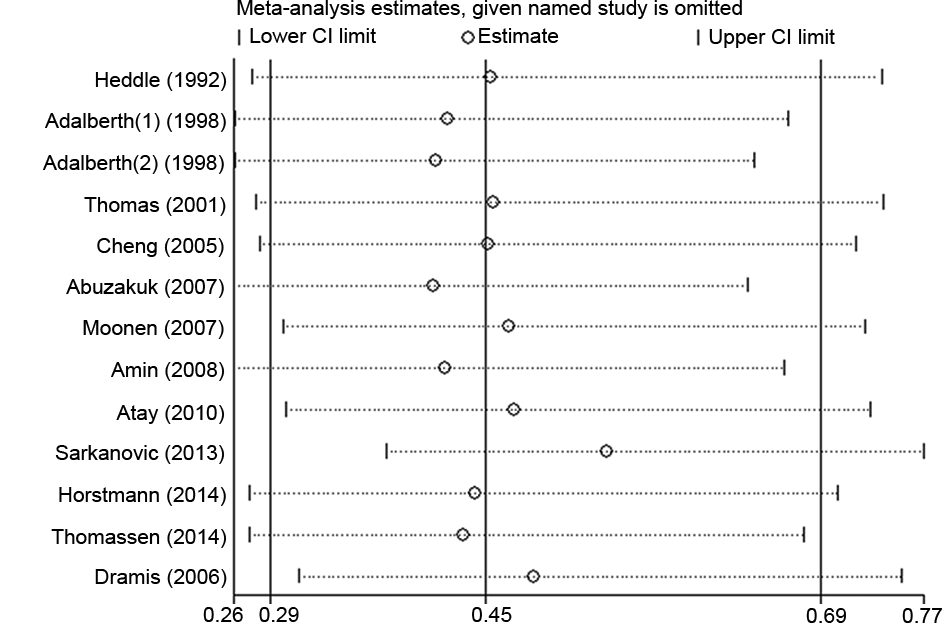

Supplement: Supplementary file 2 — Sensitivity analysis of postoperative ABT requirements in TKA. ABT, allogeneic blood transfusion; TKA, total knee arthroplasty. (TIFF 106 kb) [file 12891_2017_1710_MOESM2_ESM.tif]

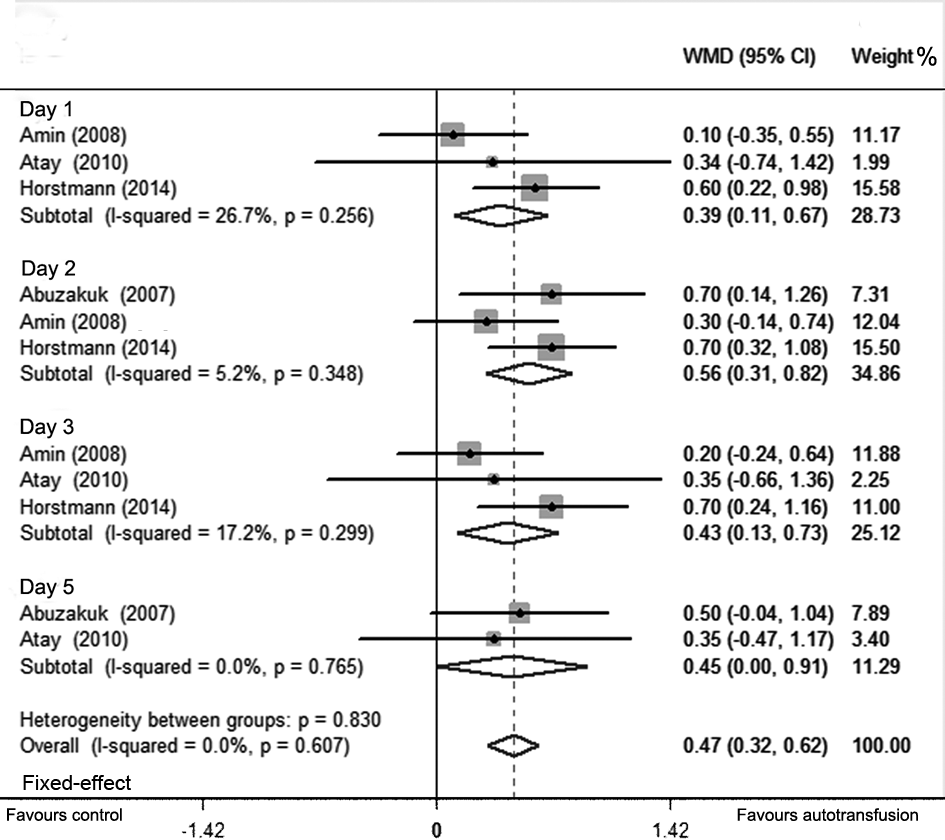

Supplement: Supplementary file 3 — Weighted mean differences (WMDs) of postoperative hemoglobin (Hb) level in TKA. TKA, total knee arthroplasty. (TIFF 190 kb) [file 12891_2017_1710_MOESM3_ESM.tif]

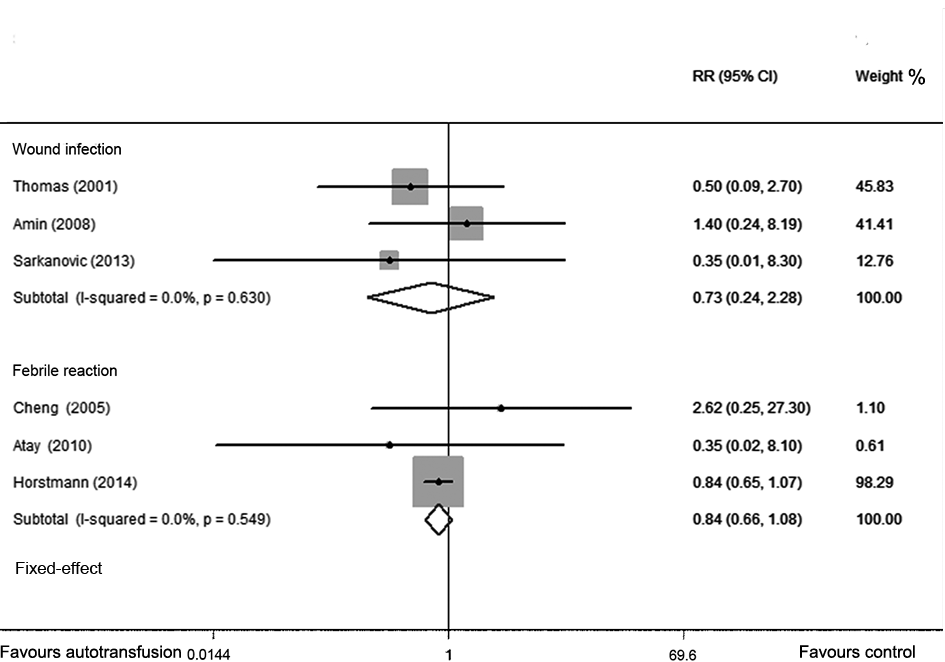

Supplement: Supplementary file 4 — Relative risk (RR) of postoperative adverse reactions in TKA. TKA, total knee arthroplasty. (TIFF 82 kb) [file 12891_2017_1710_MOESM4_ESM.tif]

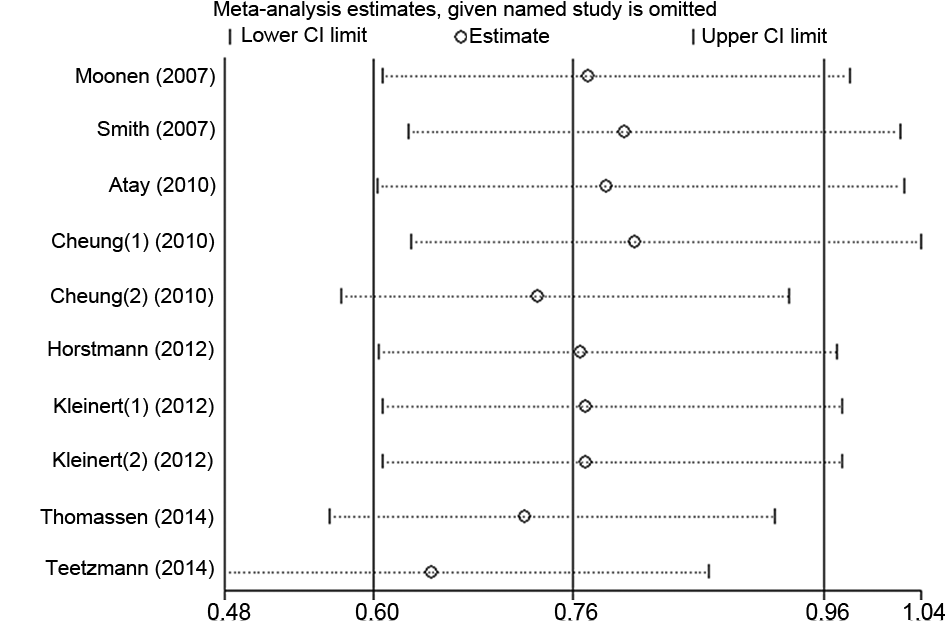

Supplement: Supplementary file 5 — Sensitivity analysis of postoperative ABT requirements in THA. ABT, allogeneic blood transfusion; THA, total hip arthroplasty. (TIFF 84 kb) [file 12891_2017_1710_MOESM5_ESM.tif]

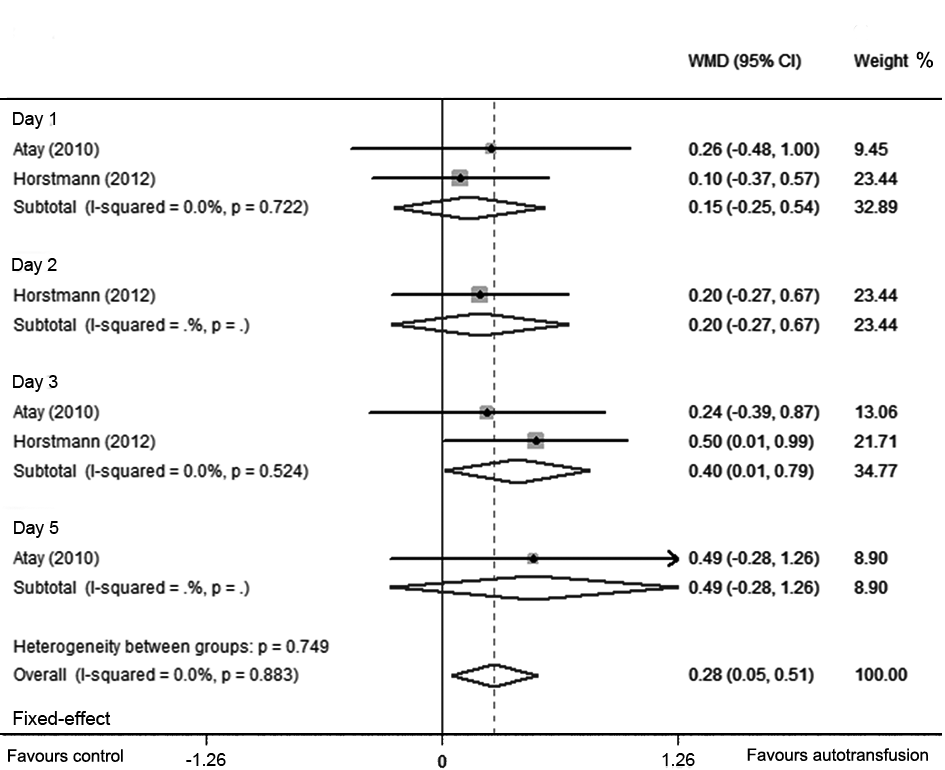

Supplement: Supplementary file 6 — Weighted mean differences (WMDs) of postoperative hemoglobin (Hb) level in THA. THA, total hip arthroplasty. (TIFF 124 kb) [file 12891_2017_1710_MOESM6_ESM.tif]

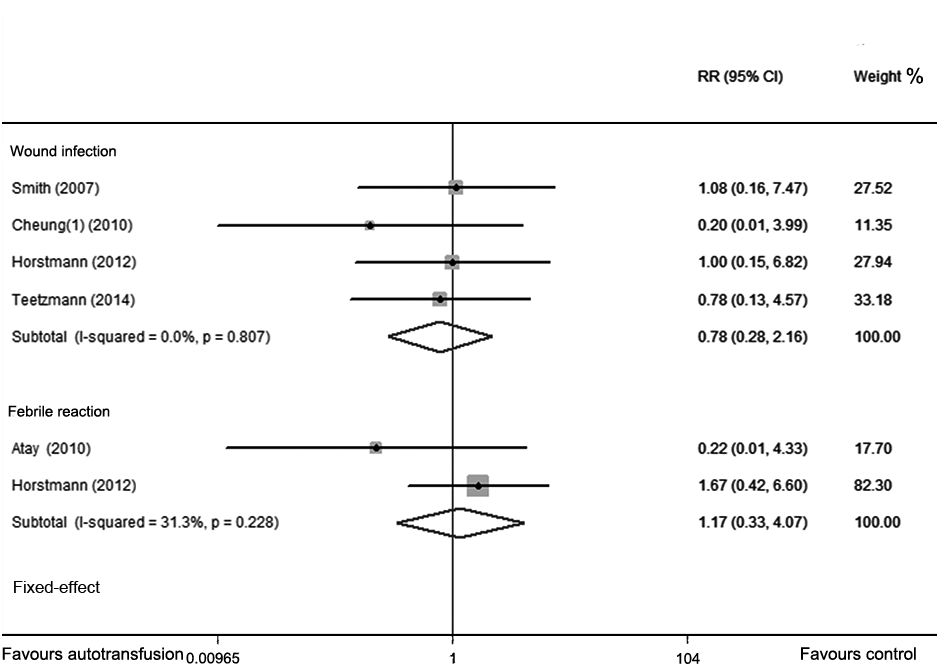

Supplement: Supplementary file 7 — Relative risk (RR) of postoperative adverse reactions in THA. THA, total hip arthroplasty. (TIFF 78 kb) [file 12891_2017_1710_MOESM7_ESM.tif]

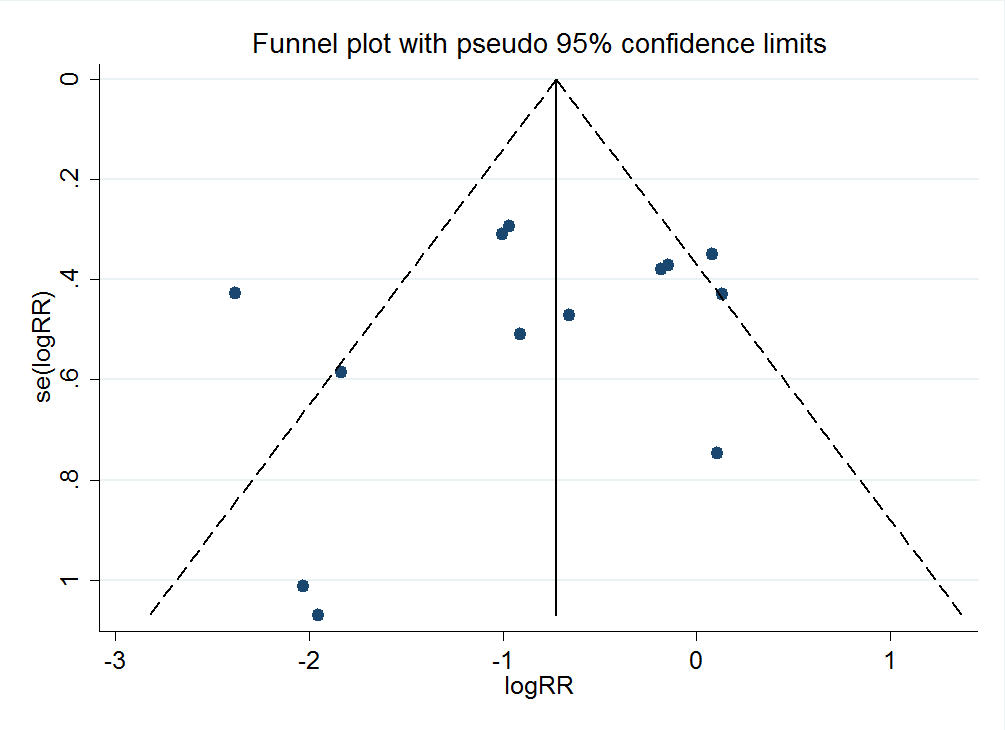

Supplement: Supplementary file 9 — Funnel plot of included studies for TKA. TKA, total knee arthroplasty. (TIFF 40 kb) [file 12891_2017_1710_MOESM9_ESM.tif]

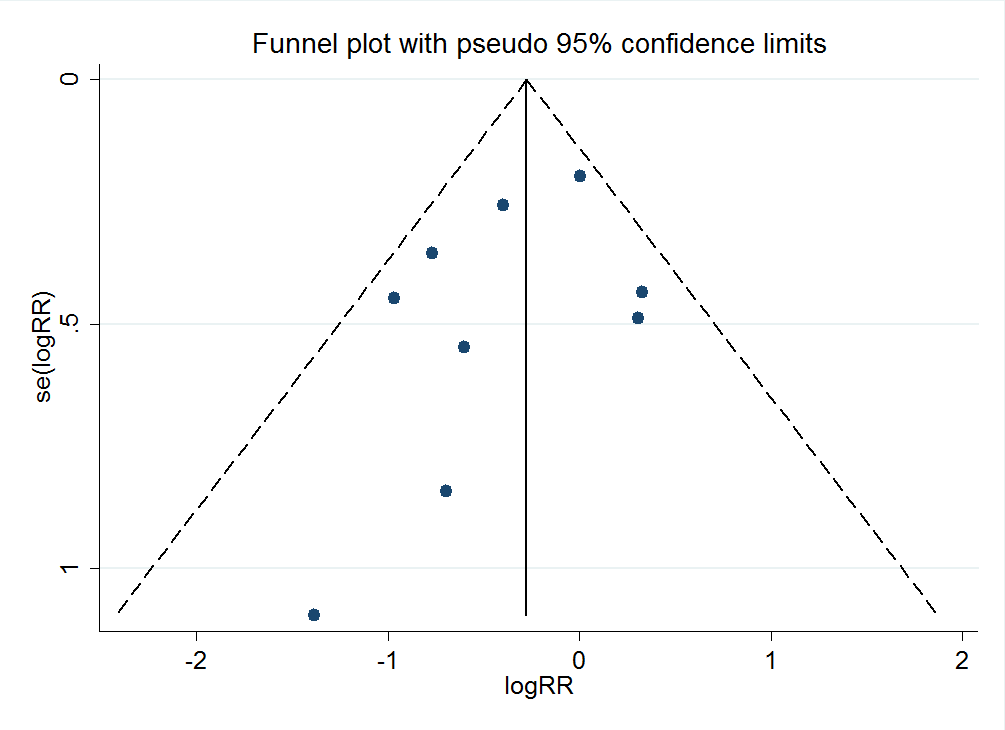

Supplement: Supplementary file 10 — Funnel plot of included studies for THA. THA, total hip arthroplasty. (TIFF 39 kb) [file 12891_2017_1710_MOESM10_ESM.tif]
