# Supplementary material for: Application of postoperative autotransfusion in total joint arthroplasty reduces allogeneic blood requirements: a meta-analysis of randomized controlled trials
Source: BMC Musculoskelet Disord. 2017 Sep 2;18:378. doi: 10.1186/s12891-017-1710-2 (PMC5581423; doi:10.1186/s12891-017-1710-2)
Supplement: Supplementary file 8 — Publication bias determined using the Egger and Begg’s tests. (DOCX 18 kb) [file 12891_2017_1710_MOESM8_ESM.docx]

| **Additional file 8: Table S1. Publication bias by the Egger' and Begg' tests** | | | | | | | | | | |
| --- | --- | --- | --- | --- | --- | --- | --- | --- | --- | --- |
|  | **Egger test** | | | | | | **Begg test** | | | |
|  | **Std_Eff** | **Coef** | **Std.Err** | **T** | **P > \|t\|** | **95% CI** | **Kendall`s Score** | **Std.Dev** | **Pr > \|z\|** | **Pr > \|z\| (continuity correct)** |
| **TKA** | **Slope** | -0.223 | 0.661 | -0.34- | 0.742 | -1.677, 1.231 | -20 | 16.39 | 0.222 | 0.246 |
|  | **Bias** | -1.236 | 1.535 | -0.81 | 0.438 | -4.615, 2.143 |  |  |  |  |
| **THA** | **Slope** | 0.098 | 0.276 | 0.35 | 0.732 | -0.539, 0.735 | -14 | 11.18 | 0.210 | 0.245 |
|  | **Bias** | -1.12 | 0.728 | -1.54 | 0.163 | -2.800, 0.561 |  |  |  |  |

Std Standard, Eff Efficiency, Coef coefficient, Err Error, CI confidence interval, Dev deviation.
