# Supplementary material for: Application of postoperative autotransfusion in total joint arthroplasty reduces allogeneic blood requirements: a meta-analysis of randomized controlled trials
Source: BMC Musculoskelet Disord. 2017 Sep 2;18:378. doi: 10.1186/s12891-017-1710-2 (PMC5581423; doi:10.1186/s12891-017-1710-2)
Supplement: Supplementary file 12 — The transfusion thresholds and funding source of included studies. (DOCX 87 kb) [file 12891_2017_1710_MOESM12_ESM.docx]

**Additional file 12: Table S2. The transfusion thresholds and funding source of included studies**

| **Study** | **Year** | **Transfusion** **threshold** | **Funding source** |
| --- | --- | --- | --- |
| **Heddle[12]** | 1992 | Haemoglobin level fell below 9 g/dl | / |
| **Adalberth[13]** | 1998 | A decrease in the hemoglobin level of more than 30% from the preoperative value or a level of less than 90g/L | / |
| **Thomas[14]** | 2001 | Haemoglobin fell below 9 g/dl | Welsh Office for Research and Development in Health and Social Care |
| **Cheng[15]** | 2005 | A hemoglobin level of less than 90g/L ,the authority of the lead physician if the patient experienced severe anaemic symptoms. | Tung Wah Group of  Hospitals Research Fund |
| **Dramis[29]** | 2006 | post-operative Hb of less than 9.0g/dl or clinical symptoms of anaemia. | / |
| **Abuzakuk[30]** | 2007 | haemoglobin count on these days was less than 9 g/dl. | / |
| **Moonen[17]** | 2007 | Depending on comorbidity classified in the ASA classification and according to hospital policy | / |
| **Smith[16]** | 2007 | Hb was less than 8.0 g/dl or if patients were symptomatic with Hb in the range of 8.0 g/dl to 10.0 g/dl. | / |
| **Amin[31]** | 2008 | Haemoglobin level fell below 8 g/dl, or if the patient developed clinical signs  of anaemia, such as tachycardia and postural hypotension, in the presence of a haemoglobin level of 8 g/dl to 10 g/dl. |  |
| **Atay[2]** | 2010 | Hemoglobin level below 8 g/dL, hematocrit level below 25% and clinical signs of anemia (tachicaridia, hypotension, dyspnea, etc.) | / |
| **Cheung[18]** | 2010 | Made by the ward doctors or anaesthetist | / |
| **Horstmann[32]** | 2012 | Hb level of 6.4 g/dl in (ASA) 1 patients, 8.0 g/dl in ASA 2/3 patients and 9.6 g/dl in ASA 4 patients and in patients that failed to increase their cardiac output to compensate for dilution | / |
| **Kleinert[33]** | 2012 | postoperative Hb was less 80 g/l or if patients were symptomatic with Hb values  in the range 80–100 g/l according to in house guidelines. | / |
| **Sarkanovic[7]** | 2013 | Haemoglobin level fell below 8.5 g/dl, | / |
| **Horstmann[24]** | 2014 | haemoglobin level of 6.4 g/dL in ASA-1 patients, 8.0 g/dL in ASA 2/3 patients,  and 9.6 g/dL in ASA 4 patients (and in patients who failed to increase their cardiac output to compensate for dilution) |  |
| **Thomassen[23]** | 2014 | Hb level exceeded 8 g/dL if the patient was symptomatic from their anaemia | / |
| **Teetzmann[22]** | 2014 | All allogeneic transfusions were prescribed by the doctor on duty according to a clinical judgement. | / |
